# Speech-in-Noise Perception in Children With Cochlear Implants, Hearing Aids, Developmental Language Disorder and Typical Development: The Effects of Linguistic and Cognitive Abilities

**DOI:** 10.3389/fpsyg.2019.02530

**Published:** 2019-11-19

**Authors:** Janne von Koss Torkildsen, Abigail Hitchins, Marte Myhrum, Ona Bø Wie

**Affiliations:** ^1^Department of Special Needs Education, Faculty of Educational Sciences, University of Oslo, Oslo, Norway; ^2^Auditory Verbal UK, Oxon, United Kingdom; ^3^Division of Head, Neck and Reconstructive Surgery, Department of Otorhinolaryngology and Head and Neck Surgery, Oslo University Hospital, Oslo, Norway; ^4^Institute of Clinical Medicine, Faculty of Medicine, University of Oslo, Oslo, Norway

**Keywords:** hearing in noise, speech in noise perception, children, hearing loss, cochlear implant, hearing aid, language ability, developmental language disorder

## Abstract

Children with hearing loss, and those with language disorders, can have excellent speech recognition in quiet, but still experience unique challenges when listening to speech in noisy environments. However, little is known about how speech-in-noise (SiN) perception relates to individual differences in cognitive and linguistic abilities in these children. The present study used the Norwegian version of the Hearing in Noise Test (HINT) to investigate SiN perception in 175 children aged 5.5–12.9 years, including children with cochlear implants (CI, *n* = 64), hearing aids (HA, *n* = 37), developmental language disorder (DLD, *n* = 16) and typical development (TD, *n* = 58). Further, the study examined whether general language ability, verbal memory span, non-verbal IQ and speech perception of monosyllables and sentences in quiet were predictors of performance on the HINT. To allow comparisons across ages, scores derived from age-based norms were used for the HINT and the tests of language and cognition. There were significant differences in SiN perception between all the groups except between the HA and DLD groups, with the CI group requiring the highest signal-to-noise ratios (i.e., poorest performance) and the TD group requiring the lowest signal-to-noise ratios. For the full sample, language ability explained significant variance in HINT performance beyond speech perception in quiet. Follow-up analyses for the separate groups revealed that language ability was a significant predictor of HINT performance for children with CI, HA, and DLD, but not for children with TD. Memory span and IQ did not predict variance in SiN perception when language ability and speech perception in quiet were taken into account. The finding of a robust relation between SiN perception and general language skills in all three clinical groups call for further investigation into the mechanisms that underlie this association.

## Introduction

Perceiving language in busy and often noisy classrooms and playgrounds is a challenge for all children in mainstream schools. The signal-to-noise ratio (SNR) is defined as the ratio between the speech dB level and the noise dB level. A negative SNR means that the noise is higher than the speech. A SNR of +15 or +20 dB is recommended for classrooms by the American Speech-Language-Hearing Association [ASHA] (1995) and the British Association of Teachers of the Deaf [BATOD] (2001), respectively. However, results from numerous studies in actual classroom situations indicate that the SNR is much lower, and in some cases even negative (Crandell and Smaldino, 1994, 2000). Not only must children listen in noisy settings, attempting to learn new words and facts, but the ambition is for better grades and better peer-to-peer relationships and social functioning. Shield and Dockrell (2008) demonstrated that external and internal noise in classrooms had a negative impact on the academic test results of typically hearing children aged 7 and 11 years. This negative relationship between performance and noise levels was maintained when the data were corrected for socio-economic factors relating to social deprivation, language, and special educational needs.

For children with hearing loss, noise makes mainstream schooling an even bigger challenge. Hearing loss affects speech-in-noise (SiN) perception via at least three main mechanisms. The first is loss of audibility, especially at high frequencies where speech sounds are lower in intensity (Soli and Wong, 2008). The second is distortion, or loss of spectral and temporal processing sensitivity and selectivity, which reduces speech perception in noise even when speech is entirely audible (Plomp, 1978, 1986; Bronkhorst, 2000). The third is less efficient binaural processing compared to typically hearing children, an essential aspect of listening in background noise, especially when speech and noise are not collocated (for a review see Bronkhorst, 2000). The latter two mechanisms will cause deficits in supra-threshold auditory processing tasks in which the audio signals of interest are audible to the listener.

In addition, there may be indirect effects of hearing loss on SiN perception via cognitive skills such as language and phonological working memory. A number of studies have shown that children with hearing loss perform less well than their typically developing peers on measures of language skills such as vocabulary and grammar (e.g., Tomblin et al., 2015; Ching et al., 2019), and on measures of phonological working memory as measured by non-word repetition or digit span tasks (e.g., Pisoni and Cleary, 2003; Lyxell et al., 2008; Davidson et al., 2019). However, the consequences of such linguistic and cognitive deficits on SiN perception are not well understood. Below we review studies that have examined the link between SiN perception and cognition generally and in children with hearing loss and language disorders specifically.

The Ease of Language Understanding (ELU) model provides a theoretical framework for how perceptual input characteristics interact with cognition in noisy listening conditions (Rönnberg et al., 2010). The model posits that as long as listening conditions are optimal, implicit processing mechanisms rapidly map the auditory input to phonological representations in long-term memory. Under noisy conditions, however, the implicit processing mechanisms may fail and lead to mismatches between input and stored phonological representations. In this situation, explicit processing mechanisms are invoked. Specifically, the listener is required to use his or her explicit working memory and linguistic knowledge to prospectively and retrospectively reconstruct the input and infer meaning. The ability to resolve mismatches between input and phonological representations will consequently depend both upon the individual’s working memory capacity and linguistic abilities. This theoretical position corresponds well with findings from a literature review by Akeroyd (2008) which examined 20 studies of the relationship between SiN perception and some aspect of cognition in mostly elderly hearing impaired adults. Results showed that measures of working memory were typically significant predictors, whereas measures of general ability, such as IQ, did not significantly predict SiN perception. However, the assumption of an association between working memory and SiN perception may hold only for some populations, such as older hearing-impaired listeners. A recent meta-analysis of studies examining young adults with typical hearing failed to find evidence of a relation between working memory (measured by the reading span task) and SiN perception (Füllgrabe and Rosen, 2016).

To our knowledge, no meta-analysis has investigated the relation between SiN perception and cognition in children, possibly because there are relatively few studies on this topic. However, the literature examining children with typical hearing suggets that the relation between SiN perception and cognitive abilities may depend on the task that is used to measure SiN perception, specifically whether it involves identification of single words or larger linguistic units. A study of school age children and adolescents by Talarico et al. (2007) found no significant correlations between SiN perception of single words and verbal or non-verbal IQ scores. In line with this, a large-scale study of school age children and young adults found no significant association between performance on the Words-in-Noise Test and receptive vocabulary (Wilson et al., 2010). On the other hand, Sullivan et al. (2015) who measured comprehension of orally presented passages, found that the relationship between auditory working memory and comprehension was stronger in noise than in quiet, indicating an increased contribution of working memory in noisy conditions. Additionally, three recent studies of SiN perception in school age children and adolescents found a significant relationship between SiN perception and (backward or forward) memory span (MacCutcheon et al., 2018, 2019, submitted). In these three studies the SiN task involved identification of two changing target words in an otherwise fixed carrier sentence. In sum, the literature to date suggests that phonological working memory and higher-order linguistic skills such as vocabulary and grammar may be more closely associated with SiN perception of sentences and passages than single words.

Studies which have focused on predictors of SiN perception for children with hearing loss specifically indicate a role for cognitive factors such as language and working memory. Ching et al. (2018) studied 252 5-year-old children with hearing aids (HA) and cochlear implants (CI) who were enrolled in the Longitudinal Outcomes of Children with Hearing Impairment study. Speech in babble perception was measured with either a word identification or sentence repetition task, depending on the language abilities of the child as judged by speech pathologists. The authors found that non-verbal IQ and language abilities were significant predictors of speech perception in babble in children using HA, with the effect size of language ability almost double that of non-verbal IQ. For children using CI, age at implantation and language abilities were significant predictors. After taking into account the effect of language ability, non-verbal IQ was not a significant predictor of speech perception in babble for children with CI. Another study by Caldwell and Nittrouer (2013) examined 27 children who used unilateral or bilateral CI, 8 children who wore bilateral HAs and 19 children with typical hearing. Children completed tasks measuring speech perception of single words in quiet and in three different SNRs (+3, 0, and −3 dB). In addition, they measured phonological awareness, general language, and cognitive skills. Children with typical hearing had better speech recognition in quiet than children with either HA or CI. However, only a small group × SNR interaction effect was observed. Interestingly, when speech perception in quiet was accounted for, there was not a significant interaction effect of group × SNR. This finding suggests that the processing limitations imposed by HA and CI had the biggest effect on recognition in quiet (when measured as phoneme score on consonant-vowel-consonant monosyllabic words), whereas the noise effects on speech perception were comparable for all children. For the participant group as a whole, general language abilities and phonological awareness explained significant variance in both phoneme and word recognition in noise. Short-term memory also explained variance in word recognition (but not phoneme recognition). However, none of these effects reached significance when the groups were considered separately.

If language, and the cognitive abilities that underlie language, do play a substantial role in SiN perception, this may also leave children with typical hearing, but a deficit in language, such as Developmental Language Disorder (DLD), vulnerable to noisy environments. Indeed, some studies have found that children with DLD have speech perception deficits in both silence and noise when tested with non-word monosyllables designed to measure discrimination of phonological contrasts (Ziegler et al., 2005, 2011). In line with this, Ziegler et al. (2011) found a significant association between SiN perception and language ability within the DLD group, but not in the typically developing control group. Another study which measured SiN perception by real word monosyllables, reported that children with DLD and co-occurring literacy impairment had a deficit in SiN perception compared to typically developing peers and children with DLD but no literacy problems (Vandewalle et al., 2012). In contrast to these results, a study by Ferguson et al. (2011) found no differences between unselected children and children with DLD in speech perception, either in quiet or in noise, when measured with both sentence lists and non-word monosyllables. However, group differences in SiN were numerically larger when measured with monosyllables than with sentences. Taken together, it appears that SiN perception may be deviant in children with DLD when tested with syllables designed to tap discrimination of phonological contrasts. However, it is unclear whether children with DLD also have a deficit when SiN perception is measured by sentences. It is well established that children with DLD exhibit a robust deficit in sentence repetition tasks when sentences are linguistically complex or long (e.g., Conti-Ramsden et al., 2001), but the sentences used in SiN tasks are typically simple and short, as the tests are constructed to measure speech perception and not general language abilities.

In addition to possibly being dependent upon cognitive abilities such as language ability and phonological working memory, SiN perception also appears to develop with age. A main finding from studies with young school-age children (5–12 years) with typical hearing is that speech perception in noise gets better during this period in development (e.g., Elliott, 1979; Fallon et al., 2000; Picard and Bradley, 2001; Jamieson et al., 2004; Stuart, 2005; Neuman et al., 2010; Wilson et al., 2010; Myhrum et al., 2016). This may be due to the protracted development of children’s auditory system, for example the gradual maturation of binaural processing (Hall and Grose, 1990; Moore et al., 2001), but also to the language development that happens during this period (Myhrum et al., 2016). This developmental trend could also be partly explained by the maturation of other cognitive abilities that may be involved in SiN perception, such as attention and processing speed (Gomes et al., 2000; Luna et al., 2004).

Over the last 25 years, research has devised a number of adaptive test paradigms, using words or sentences and different maskers, to measure SiN perception. Examples of such tests include the Hearing in Noise Test (HINT; Nilsson et al., 1994), the Speech Recognition in Noise Test (SPRINT, Cord et al., 1992), and the Words-in-Noise Test (WIN; Wilson, 2003). The HINT is one of the most widely used adaptive tests of SiN perception (Harianawala et al., 2019). It is commonly used in clinical practice, and the paper by Soli and Wong (2008) lists normative data for 13 different languages. The developers of the HINT have attempted to address many of the factors which are known to affect SiN, such as the speech material, masking noise and test room acoustics (Soli and Wong, 2008). For example, the speech materials consist of “Short, simple sentences from children’s books at a first grade reading level” (Soli and Wong, 2008, p. 356). The sentences are evaluated for naturalness by native speakers, and those with low scores are rejected or modified. When children’s versions of the HINT are developed, sentences are also evaluated specifically on appropriateness for the youngest school age children (Myhrum et al., 2016). Still, the previous literature reviewed above suggest that it is possible that the sentences which are acceptable for 5-year-old typically developing children may be challenging in terms of linguistic complexity, cognitive or working memory demands for children with hearing loss or developmental disabilities, especially when presented in noise. This is a critical question, as these are the target populations for the HINT in clinical practice. It will therefore be useful to know whether language skills, working memory or non-verbal abilities may explain variance in performance on the HINT for children with hearing loss or language disorders.

The main aims of the present study were firstly, to investigate the differences in SiN perception, as measured by the HINT, between four groups of school-age children—children using CI, children using HA, children with DLD and typically developing (TD) children. Secondly, the study aimed to explore whether language ability, working memory or non-verbal IQ could explain variance in HINT performance.

## Materials and Methods

### Participants

The 175 participants in the present study were recruited from a wider project to specifically investigate performance on the HINT in children aged 5.5–12.9 years old. The wider project was approved by the Regional Committees for Medical and Health Research Ethics, South-East Norway. Written informed consent was obtained from the parents of all participants. In addition, oral consent was obtained from the participating children after receiving information about the study and the tasks involved.

The inclusion criteria in the present study were set to investigate performance on the HINT and control for other factors that could affect performance on the HINT. All children, in the CI, HA, DLD and TD groups, met the following inclusion criteria: (1) They had completed the HINT, (2) they all had a standard score of 75 or above on the non-verbal IQ test Raven’s Progressive matrices (Raven et al., 2004; Raven and Raven, 2008), (3) the child, and at least one of the child’s parents, had spoken Norwegian as their native language, and (4) no diagnosis of other developmental disorders such as autism or ADHD had been made. An additional inclusion criterion for the TD and DLD groups was (5) parent report of normal hearing and the presence of otoacoustic emissions (OAEs) in both ears, indicating no damage in outer-hair-cell function. Presence of OAEs is in most cases associated with normal hearing sensitivity and hearing thresholds (Lucertini et al., 2002; Engdahl et al., 2005; Stach, 2010). An additional inclusion criterion for the CI and HA groups was (6) the child used bilateral HAs or CIs. There were two additional criteria, (7) and (8), used in the recruitment of the DLD group. These are detailed under ‘Characteristics of the children in the DLD group’ below.

In the present study, no official diagnosis of additional needs were made. Criterion 2 was set to prevent including children who had intellectual disabilities, defined in DSM V as IQ scores below 70, including a margin of measurement error (American Association on Intellectual and Developmental Disabilities, 2010; American Psychiatric Association, 2013). This criterion adhered to the definition of DLD that language difficulties should not be associated with known biomedical conditions such as intellectual disabilities (Bishop et al., 2017).

**FIGURE 1:**
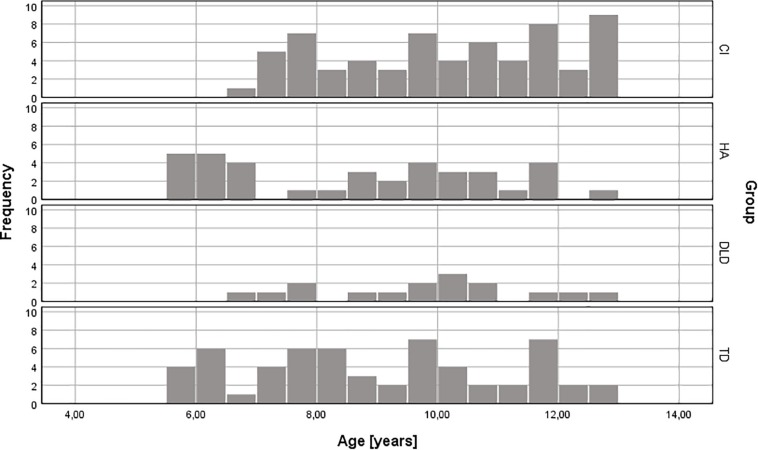
Age distribution in the four participant groups.

Participants in the present study included 64 children using bilateral cochlear implants (CI) (38 boys, 59%), 37 children using bilateral hearing aids (HA) (16 boys, 43%), 16 children with DLD (11 boys, 69%), and 58 children with typical development (TD) (22 boys, 38%). Children in all groups were recruited in the same age range (5.5–12.9 years), and the groups had a similar age distribution (see Table 1 and Figure 1). However, the groups were not matched for age. A one-way ANOVA showed a significant effect of group on age [*F*(3,171) = 5.3, *p* = 0.002]. *Post hoc* comparisons using the Bonferroni correction, showed that there were significant differences in age between the TD and CI groups (*p* = 0.013) and the HA and CI groups (*p* = 0.004). The other pairwise comparisons had *p*-values equal to 1.0 except for the group comparison between HA and DLD (*p* = 0.48). Results on the HINT and cognitive tests were adjusted for age, where appropriate, to enable adequate comparison between groups (see section Test Materials and Procedure for details).

**TABLE 1:** Descriptive statistics by group for age, speech perception, language ability, non-verbal IQ, and memory span.

	**CI (*n* = 64)**	**HA (*n* = 37)**	**DLD (*n* = 16)**	**TD (*n* = 58)**
				
	**Mean (*SD*) (range)**	**Mean (*SD*) (range)**	**Mean (*SD*) (range)**	**Mean (*SD*) (range)**
Age [years]	10.1 (1.8) (6.9–12.9)	8.7 (2.2) (5.5–12.7)	9.7 (1.8) (6.5–12.5)	9.0 (2.0) (5.7–12.8)
Speech perception (monosyllables)	87.2 (7.0) (68–100)	88.4 (11.0) (48–100)	95.6 (8.4) (70–100)	99.0 (1.6) (92–100)
Speech perception (sentences)	96.3 (5.6) (77–100)	95.8 (6.0) (70–100)	93.6 (16.1) (42–100)	99.7 (0.8) (96–100)
Speech-in-noise perception (SiN)	2.6 (2.5) (−3.2–10.5)	0.6 (3.1) (−3.7–13.6)	−0.8 (2.3) (−4.1–4.4)	−2.9 (1.2) (−5.4–−0.3)
Language ability	76.1 (18.4) (42–114)	85.2 (15.5) (47–117)	66.9 (15.7) (44–102)	102.7 (14.9) (57–135)
Non-verbal IQ	97.7 (11.2) (75–120)	98.5 (16.1) (75–135)	90.6 (11.8) (75–115)	103.6 (13.6) (80–145)
Memory span	6.3 (2.0) (2–11)	7.8 (2.4) (2–13)	5.3 (2.4) (2–10)	8.5 (2.2) (4–15)

*SD, standard deviation; Speech perception (monosyllables) (% correct words); speech perception (sentences) (% correct words); SiN perception measured by age-adjusted HINT SRT in noise (dB SNR); language ability measured by The Core Language Index of the Clinical Evaluation of Language Fundamentals (CELF-4) (standard score); non-verbal IQ measured by Raven (standard score); memory span measured by CELF Digit span subtest (scaled score).*

#### Characteristics of the Children in the CI Group

Participants in the wider project were recruited from a clinical population with a wide range of hearing abilities. In this current smaller study, which focused on SiN perception measured by the HINT (sentence repetition), participants were included only if they had hearing abilities which were good enough to repeat sentences presented in quiet. Consequently, the children with CI included in the present study were those with relatively good speech perception, and they are thus not representative of the whole pediatric CI population.

The onset of hearing loss was reported, in medical journals, as before 12 months of age for the majority (81%) of children with CI (see Table 2). All children received their first CI (in either sequential or simultaneous bilateral implantation) between November 2002 and December 2014. Amongst the children whose onset of hearing loss was prior to 12 months, 24% were implanted by 12 months of age, 37% were implanted between 1 and 2 years of age, and 39% were implanted after the age of 2 years. There were 26 children who were implanted after 3 years of age, and six of these had an onset of hearing loss prior to 12 months of age. The remaining children either had normal hearing or some residual hearing after birth. In the present study the youngest children to receive CIs were 5 months old. To investigate the effect of implantation age, the participants in the CI group were classified as either (1) having acquired oral language before implantation (*n* = 18) or (2) having acquired no or very little oral language before implantation (*n* = 46). This classification was made based on medical journals and parental report. It was considered relevant to investigate the effects of implantation age only for children in the second subgroup.

**TABLE 2:** Characteristics of the CI and HA groups.

	**CI group**	**HA group**
	**(*n* = 64)**	**(*n* = 37)**
**Onset of hearing loss**		
<12 months	81%	76%
>12 months or unknown	19%	24%
**Communication approach**		
Spoken language	80%	78%
Spoken language with some sign support	11%	11%
Spoken language with lots of sign support	1%	–
Missing data	8%	11%
**Educational setting**		
Mainstream	92%	95%
Hearing unit in mainstream school	3%	3%
School for children with hearing loss	5%	2%

In Norway, the cost of CI implantation is covered by the government and bilateral implantation is offered as the standard procedure for children under 18 years. All children in this study wore bilateral CIs. Fifty-five percent were implanted simultaneously and 45% were implanted sequentially.

The make of implants used by participants were Cochlear (55%) and MED-El (45%). Among the 35 children using Cochlear devices, 89% (31 children) were fitted bilaterally with the same model: CI24R/RE (27 users) and CI512 (four users). The remaining four children with Cochlear devices had different models implanted in the right versus the left ear with a combination of CI24RE and CI422 (one user), CI24R/RE and CI512 (two users), CI513 and CI512 (one user). Among the 29 children using MED-EL devices, 69% (20 children) were fitted bilaterally with the same model: C40 + (one user), CONCERTO FLEX24 (one user), PULSARci100 (11 users) and with SONATti100 (seven users). The remaining nine children with MED-EL devices had different models implanted in the right versus the left ear with a combination of PULSARci100 and C40+ (five users), PULSARci100 and CONCERTO (one user), and PULSARci100 and SONATti100 (three users). The use of different models in the right versus the left ear can be explained by the date of implantation, and that the newest available CI model was implanted if the child received sequential CIs or in cases of reimplantation.

The cause of hearing loss was identified for 67% of the children with CI, with a genetic abnormality for the Connexin 26 protein being the most common etiology (29% of the children). Other common causes of hearing loss were Pendred or LVAS (Large Vestibular Aqueduct Syndrome) (16%) and meningitis infection (19%). For communication approach and educational settings of participants with CI, see Table 2.

#### Characteristics of the Children in the HA Group

The onset of hearing loss was reported, by parents, as before 12 months of age for the majority (76%) of children (see Table 2). According to the descriptors recommended by the World Health Organization (WHO), two children (5%) had a mild hearing loss, 27 (73%) had a moderate hearing loss, 7 children (19%) had a severe hearing loss, and 1 child (3%) had profound hearing loss in the better hearing ear. The majority of children (87%) had symmetric hearing loss with a difference of less than 10 dB hearing threshold levels between the ears according to Pure Tone Average (PTA) (measured at frequency 500, 1000, 2000, and 4000 Hz). Five children had PTA differences of 10, 11, 17, 22, and 60 dBHL respectively. All children used bilateral HAs. The children were tested in conjunction with their regular hearing device checkups at the Ear-, Nose and Throat clinic at their local hospital. The assessment session was carried out after the device checkup and adjustment, thus securing that the child had well-functioning hearing devices at the time of assessment. For communication approach and educational settings of participants with HA, see Table 2.

#### Characteristics of the Children in the DLD Group

The participants with DLD comprised a clinical sample which was recruited from the educational and psychological counseling service in municipalities across Norway. This service has the responsibility for the assessment and counseling for children with developmental difficulties in Norway. In addition to the general inclusion criteria 1–5 reported above, two additional inclusion criteria for the DLD group were, (6) referral to the educational and psychological counseling service for language difficulties, and to independently confirm the status as developmentally language disordered, (7) scores 1 SD or more below the normative mean on at least two out of the following five standardized tests: The British Picture Vocabulary Scale II (BPVS-II; Dunn et al., 1997; Lyster et al., 2010), the children’s test of non-word repetition (Gathercole et al., 1994; Norwegian version by Furnes and Samuelsson, 2009), and three subtests from the Clinical Evaluation of Language Fundamentals (CELF-4; Semel et al., 2003): Concepts and Following Directions, Formulated Sentences, and Recalling Sentences. One child who was recruited in the DLD group completed all standardized tests, but did not satisfy criteria 7 and so was excluded, leaving 16 children with DLD in the sample. All the children were fully integrated into mainstream school education.

#### Characteristics of the Children in the TD Group

All of the children with TD were recruited from mainstream educational settings across Norway. Children in this group were defined as ‘typically developing’ by their teachers.

### Test Materials and Procedure

Standard scores which were derived from age-based norms were used for language and cognitive tests, and age norms were used to adjust HINT scores based on normative data (see description in section Speech-in-Noise Perception). The three tests of speech perception were always administered in the same order: (1) perception of monosyllables in quiet, (2) perception of sentences in quiet, and (3) perception (of sentences) in noise. Except for this, the order of the tests was randomized. All tests were administered in a one-to-one setting in a quiet room. All the participants with hearing loss wore their hearing devices bilaterally during the entire test session, including both the speech perception tasks and the cognitive/linguistic tasks.

#### Speech Perception in Quiet: Monosyllables

The Norwegian Phonetically Balanced word lists consisting of 50 monosyllabic words each (Øygarden, 2009) were presented from a speaker 2 m in front of the listener at 65 dBA. The main objective of this test was to assess the children’s ability to discriminate speech sounds. The monosyllables are Norwegian words with a high usage frequency, but as they are monosyllabic, they are difficult to guess if not all of the speech sounds are identified correctly. Some of the words differ from other Norwegian words by a single phoneme. The child’s score represented the percentage of words that were repeated correctly.

#### Speech Perception in Quiet: Sentences

The HINT sentences (for description, see Speech-in-Noise Perception below) in quiet were presented at 65 dBA. The number of words the child was able to repeat accurately were counted to calculate a percentage score of words in sentences. The sentence repetition test in quiet served two purposes: (1) to measure speech perception in quiet, and (2) as a pretest to the HINT (in noise) to familiarize the child with a test which requires repetition of sentences.

#### Speech-in-Noise Perception

SiN perception was assessed with the Norwegian HINT for children (NHINT-C). For the sake of brevity, we refer to the NHINT-C as HINT in this paper. The adaptive procedure described in Soli and Wong (2008) was used to estimate the speech reception threshold (SRT) in speech-shaped noise fixed at 60 dBA. The SRT was defined as the mean SNR at which the listener could repeat 50% of the sentences correctly (ignoring inflectional errors and additional words). The SiN performance was evaluated under the condition where speech and noise came from a speaker (Sony SS-MB150H) one meter in front of the participant. Participants were presented with two lists each composed of ten sentences that they were asked to repeat. The speech levels were adjusted for each sentence depending on whether the previous sentence was repeated correctly or not (thereby the name adaptive procedure). The SRTs of the two lists were averaged. HINT SRTs were adjusted for age and room effects as described below, to yield the final measure of SiN perception.

The principle behind the HINT is that the sentences used in the test should be short, the syntax simple, and the vocabulary familiar even to preschool children (Soli and Wong, 2008). Thus, the linguistic and memory demands of the sentences are assumed to be minimal. As reported by Myhrum et al. (2016) the sentences used in the Norwegian child version were a subset (120 sentences) of the 240 sentences of the Norwegian HINT for adults. The sentences included in the child version were selected in a two-step process: First five adult raters, including three pediatric speech and language pathologists selected 158 of the 240 sentences, which were judged to be comprehensible and repeatable for 5–6-year-old children. Second, the sentences were tested on 11 TD children aged 4.8–5.6 years. The 120 sentences with the highest accuracy scores were identified and divided into 12 phonemically matched 10-sentence lists. Trial-and-error was used to adjust the composition of the lists to obtain the closest match of their phoneme distributions to the overall distribution. The average length of the 120 included sentences was 5.2 words (*SD* = 1.0, range 3–8).

In the previous study by Myhrum et al. (2016) with typically hearing children from 5;6 years, all the children who were tested with the sentences in quiet performed at ceiling. As a rule of thumb in clinical practice, the word score of sentences in quiet must be above 75% for the child to be tested with the sentences in noise.

For children with typical hearing, HINT results depend on age. In order to know how a child performed compared to a population of normal hearing children of the same age, the normative mean value of his or her age group was subtracted from each HINT SRT. Norwegian HINT normative data across ages from 5;6 to 13;0 years of age were reported in a study described by Myhrum et al. (2016), and the linear regression equation for the age-specific correction for age 5;6 to 10;5 years was used to calculate age-specific correction factors in the current study. HINT results of children older than 10;4 years were not adjusted for age. By adjusting for age, the age effect observed in the normative HINT SRT material is taken into account, and the age-adjusted SRTs can be used as in analyses together with standard scores from the other tests used in the study.

Participants were tested in mainly one room, the anechoic chamber used in the normative study (Myhrum et al., 2016). However, due to large geographical distances from the clinic, 21 participants were tested in two other audiometric test rooms. Since HINT results obtained in a sound field will be influenced by room acoustics, Nilsson et al. (1996) proposed age-specific correction factors relative to adult performance to allow comparison across different sound fields. This was described as a five step procedure in Vaillancourt et al. (2008): (1) obtain adult norm in sound field A, (2) obtain age-specific normative data in sound field A, (3) calculate age-specific correction factors from step one and two, (4) obtain adult norm in sound field B, and (5) calculate age-specific norms for sound field B which are the sums of the age-specific correction factors (3) and the adult norm (4).

The anechoic chamber used in the normative study was defined as sound field A with HINT adult norm −3.9 dB SNR, and the two other audiometric rooms were defined as sound field B1 and sound field B2. The adult norm in sound field B1 was calculated as the average SRT (−2.6 dB SNR) obtained from ten normal hearing adults. Five children from the TD group and eight from the HA group were tested in sound field B1, and their HINT SRTs were corrected for the room effect by −1.3 dB. The adult norm in sound field B2 was not collected, and the HINT SRTs were not corrected for the room effect. Eight children from the HA group were tested in sound field B2.

#### Language Ability

General language ability was measured by the Norwegian adaptation of the Clinical Evaluation of Language Fundamentals 4 (CELF-4; Semel et al., 2003). CELF-4 is a comprehensive test of language skills, consisting of 13 subtests measuring different aspects of expressive and receptive language as well as verbal memory. There are two slightly different versions of the CELF: one for children aged 5;5–8;9 years and one for children aged 9–12;11 years. The Core Language Index (CLI) is the main index of the test and is intended to be a measure of general language ability that can be used to make decisions about whether a child has a language disorder or not. The CLI for children aged 5;5–8;9 years comprises the following four subtests: Concepts and Following Directions, Word structure, Recalling Sentences and Formulated sentences. The CLI for children aged 9;0–12;11 years comprises the same subtests except that Word Structure has been replaced with Word Classes 2 Total. The Concepts and Following Directions subtest measures the child’s ability to follow oral directions of increasing length and complexity by pointing to one or more pictured objects in the correct order. The Word Structure subtest examines the child’s morphological knowledge (e.g., plurals and past tense conjugations) by asking the child to complete orally presented sentences in reference to a picture. In the Recalling Sentences subtest the child is asked to repeat orally presented sentences. In the Formulated sentences task, the child is asked to generate sentences in response to orally presented words and a pictures. In the Word Classes 2 task, the child is given three to four orally presented words and is asked to (1) identify two words among these that go together and (2) explain their relationship. We used the CLI (which is a standard score derived from age norms) in all statistical analyses reported below. The CELF-4 has been normed with a sample of 600 Scandinavian children aged 5;0–12;11 years to give the normal range 86–115.

#### Non-verbal IQ

General non-verbal IQ was measured by Raven’s Colored Progressive Matrices for children aged 5;5–8;11 years and Raven’s Standard Progressive Matrices (standard version or plus version) for children aged 9;0–12;11 years (Raven et al., 2004). Both tests consist of a series of visual patterns with one part of the pattern missing. The child is presented with a number of options and is instructed to select the correct part to complete the designs. The standard score for non-verbal IQ (derived from age norms) was used in the analyses reported below. The normal range is 86–115.

#### Memory Span

Memory span was measured by the digit span subtest from the Norwegian adaptation of the CELF-4 (Semel et al., 2003). The child is asked to repeat sequences of orally presented numbers of increasing length and difficulty, either in the order they are presented or backwards, starting with two numbers in sequence and ending (if stop criteria are not applied earlier) with a sequence of nine numbers. Stop criteria are set at two incorrect repetitions of sequences of the same length and difficulty. A score of one was given for each correctly repeated sequence and a score of zero for each incorrectly repeated sequence. Scores obtained in the forward and the backwards repetition tasks were summed, and the highest possible score was 30 (16 points for the forward and 14 points for the backwards repetition). The raw score was transformed into a scaled score according to the age-based norm given in the CELF-4 manual. The scaled score from this test was used in the regression and correlation analyses reported below. The normal range was 7–13 with 10 as normative mean and 3 as + −1 standard deviation.

### Analysis

The statistical analyses were carried out in SPSS for Windows v.25 (SPSS Inc., 2018). SiN perception scores were adjusted for age using the linear regression of age norms calculated in Myhrum et al. (2016). Standard scores derived from age norms were used for language ability and non-verbal IQ, and scaled scores were used for memory span. Speech perception of monosyllables and sentences in quiet were not adjusted for age as these measures are designed to be mastered by children aged 5–6 years, e.g., in Myhrum et al. (2016) normal hearing children of age from 5;6 years old were tested with HINT in quiet sentences and scored 100%. All variables used in the analyses were therefore expected to be independent of age, since the values represent performance compared to a norming sample of the same age. This means that if 6-year-old and a 10-year-old both obtain a standard score of e.g., 75 for language ability, they are both equally behind their age peers, but the actual language skills of the 10-year-old are more advanced than a the actual language skills of the 6-year-old. A parallel example for the age corrected HINT SRTs is that a 6-year-old with an age-corrected SRT of 2 dB will actually have a higher SRT than a 10-year-old with the equal age-corrected SRT of 2 dB, but the two children perform equally in comparison to their normal-hearing peers.

For monosyllable and sentence perception in quiet, there was a ceiling effect and a small range of variance in the two groups with typical hearing, the TD and DLD groups. One-way analyses of variance (ANOVAs) were used to test for differences between groups in SiN perception, language ability, non-verbal IQ and memory span. *Post hoc* comparisons used the Bonferroni correction. However, the *post hoc* comparisons were also carried out using the Hochberg’s GT2 and Games-Howell tests to account for the differences in sample sizes and, on occasion, unequal variances (Field, 2013), but the use of those tests did not change any of the significant findings. Thus only the comparisons using the Bonferroni correction are reported.

To investigate which variables influence SiN perception (measured by SRTs adjusted for age), data were first analyzed in one regression model with all participants where group was added as one of the predictors (using groups as independent binary dummy variables). Pearson correlations were calculated to measure associations between SiN and the independent variables. In addition to investigating predictors to SiN in the full dataset, follow-up multiple regression models were fitted separately to data for children with CI, children with HA and children with TD. Simple linear regression is reported for the DLD group as the sample size was too small to perform multiple regression analysis. Diagnostic statistics, such as Cook’s distance, Mahalanobis distance and the DFBeta statistics, were used to assess the fit of the regression models and identify any influential points that were having any undue influence on the model (Barnett and Lewis, 1978; Cook and Weisberg, 1982; Stevens, 2002).

## Results

### Group Differences

#### Speech Perception in Quiet (Monosyllables) for Children With CI, HA, DLD, and TD

Table 1 shows descriptive statistics of the monosyllable scores for children with CI, HA, DLD, and TD. In the TD group, 36 children (62%) scored 100% on the monosyllable perception test, 18 children scored 98% and 4 children scored between 92 and 96%. In the DLD group, the monosyllable perception scores were above 95% for all participants except for two participants who scored 70 and 80% respectively. Thus, the participants in the TD and DLD groups had a close to perfect recognition of monosyllables, compared to participants in the HA and CI groups who scored on average 88 and 87% respectively.

#### Speech Perception in Quiet (Sentences) for Children With CI, HA, DLD, and TD

Table 1 shows descriptive statistics for the speech perception of sentences in quiet for children with CI, HA, DLD, and TD. In the TD group, fifty children (86%) scored 100% on this test, 7 children scored 98% and 1 child scored 96%. In the DLD group, all scores were above 98% except for two participants who scored 66 and 42% (these were the same two participants who scored 70 and 80% on the monosyllables test). Thus except for those two children, all participants in the TD and DLD groups repeated the ten sentences in the speech perception in quiet test without errors or with only a single error.

In the CI group, 53% (*n* = 34) and in the HA group, 38% (*n* = 14) repeated all sentences correctly, and 89% of the participants in the HA and CI group had a score of 90% correct or better. This leaves only 11% (7 in the CI group and 4 in the HA group) with scores below 90%.

#### Speech-in-Noise Perception for Children With CI, HA, DLD, and TD

**FIGURE 2:**
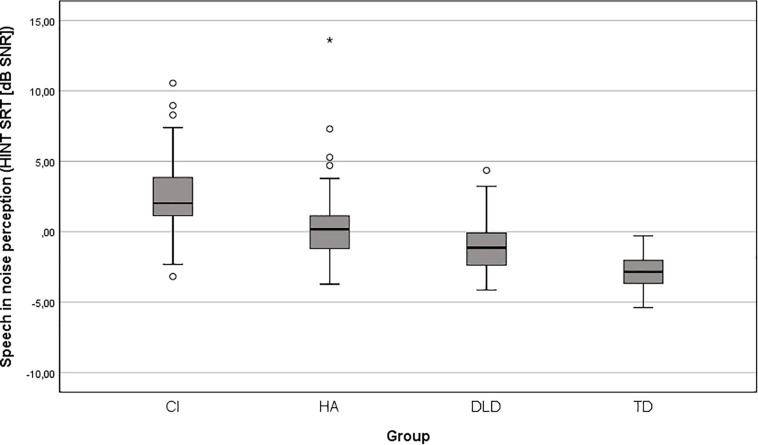
Boxplot of age-adjusted HINT SRTs for children with CI, HA, DLD, and TD. The boxes go from the first quartiles to the third quartiles. Outliers were defined using Tukey (1977) i.e., as any data points more than 1.5 times the interquartile range from the quartiles. The whiskers go from the smallest non-outlier to the largest non-outlier.

Figure 2 shows a boxplot of SiN perception scores for children with CI, HA, DLD, and TD with outliers displayed as dots. On average, children with CI had 2.0 dB higher SRTs than children with HA, 3.4 dB higher SRTs than children with DLD and 5.5 dB higher SRTs than children with TD (see Table 1). A one-way ANOVA showed a significant effect of group on SRT, [*F*(3,171) = 59.4, *p* < 0.001, η^2^ = 0.51]. *Post hoc* comparisons, using the Bonferroni correction, revealed that there was no difference between the HA and DLD groups (*p* = 0.20) and significant differences in mean SRT between the other groups [*p* < 0.001 for all, except for between the TD and DLD group (*p* = 0.01)].

#### Language Ability for Children With CI, HA, DLD, and TD

Table 1 shows descriptive statistics for the CELF CLI for children with CI, HA, DLD, and TD. Seven children in the TD group scored below the normal range for the CLI, and two children in the DLD group scored within the normal range on this index. As the definition of DLD relies on a deficit in functional language ability, e.g., affecting everyday and school functioning and a complete clinical assessment, the TD children were not excluded despite being outside of the normal range, as they had been defined as ‘typically developing’ by their teachers. The result of one assessment is not sufficient to confirm language difficulties in these children. Similarly, the DLD children who scored within the normal range on the CLI were not excluded, as they still met criteria 6 and 7 (see section Characteristics of the Children in the DLD Group). Furthermore, in the HA group 4 children (11%) scored below −2 SD from the normative mean, and in the CI group 24 children (38%) scored below −2 SD from the normative mean. There was a significant effect of group on language ability [*F*(3,171) = 34.80, *p* < 0.001, η^2^ = 0.38]. On average, children with TD scored within the normal range for the CLI (86–114) (Semel et al., 2003), children with HA scored just below this range, children with CI scored on average below −1 SD from the normative mean, and children with DLD scored on average below −2 SD from the normative mean. *Post hoc* comparisons, using the Bonferroni correction, revealed that there were significant differences in language ability between all groups at the *p* < 0.001 level, except between the HA and CI groups (*p* = 0.049) and between the CI and DLD groups (*p* = 0.29).

#### Non-verbal IQ for Children With CI, HA, DLD, and TD

Table 1 shows descriptive statistics of the non-verbal IQ scores for children with CI, HA, DLD, and TD. There was a significant effect of group [*F*(3,169) = 4.7, *p* = 0.004, η^2^ = 0.08]. *Post hoc* comparisons, using the Bonferroni correction, showed that there was a significant difference of 13.0 in non-verbal IQ scores between the TD and DLD groups (*p* = 0.004). There was no significant difference in mean non-verbal IQ scores between any other groups at *p* < 0.05 (TD and HA (*p* = 0.42), TD and CI (*p* = 0.09), HA and DLD (*p* = 0.29), HA and CI (*p* = 1.0), CI and DLD (*p* = 0.36)].

#### Memory Span for Children With CI, HA, DLD, and TD

Table 1 shows descriptive statistics for memory span for children with CI, HA, DLD, and TD. A one-way ANOVA showed a significant effect of group on digit span scores [*F*(3,171) = 16.7, *p* < 0.001, η^2^ = 0.23]. *Post hoc* comparisons, using the Bonferroni correction, showed that there were significant differences (*p* < 0.005) in mean digit span scores in all pairwise group comparisons, except no significant differences between the DLD and CI groups (*p* = 0.60) or between the TD and HA groups (*p* = 0.61).

### Regression Analyses

Since we used standardized scores derived from age-norms for the independent variables and age-corrected scores (based on age norms) for the dependent variable, age was not included in the regression models. The regression model was first run on the full sample of 175 children. Subsequently, the effect of the predictor variables were explored by running linear models for each of the separate groups.

#### Regression Analysis Using Data From All Groups

Table 3 reports correlations among all variables in the full sample. In the regression model predicting SiN perception with only group and language ability as predictors, the model explained 60% (*R*^2^ = 0.60) of the variance (*F* = 64.5, *p* < 0.001). The mean values for SiN perception for each group changed when adjusting for language ability, and the mean values for SiN perception were no longer significantly different between the DLD group and the TD group (*p* = 0.87). The regression coefficient of language ability was −0.061 (*p* < 0.001) when included in the model together with the group variable. When speech perception in quiet (monosyllables) was added to the model, it explained 65% (*R*^2^ = 0.65) of the variance in SiN perception, and the regression coefficients of language ability and monosyllable perception were significant predictors and equal to −0.057 and −0.094 respectively. This means that a 10 point increase in language ability was associated with a 0.6 dB decrease in SiN and that a 10 point increase in speech perception in quiet (monosyllables) was associated with a 0.9 dB decrease in SiN. When adding non-verbal IQ and memory span to the model, this did not explain any more of the variance in SiN perception. Table 4 shows the results of the multiple linear regression model for SiN perception with all the explored variables included. The model accounted for 64% of the variance.

**TABLE 3:** Correlations among variables for the full group of participants (*n* = 175).

	**Speech in noise perception (SiN)**	**Speech perception (sentences)**	**Speech perception (monosyllables)**	**Language ability**	**Non-verbal IQ**	**Memory span**
Speech in noise perception (SiN)	1	−0.47^∗∗^ *p* < 0.001	−0.60^∗∗^ *p* < 0.001	−0.60^∗∗^ *p* < 0.001	−0.27^∗∗^ *p* < 0.001	−0.33^∗∗^ *p* < 0.001
Speech perception (sentences)	−0.47^∗∗^ *p* < 0.001	1	0.40^∗∗^ *p* < 0.001	0.45^∗∗^ *p* < 0.001	0.16^∗^ *p* = 0.037	0.23^∗∗^ *p* = 0.002
Speech perception (monosyllables)	−0.60^∗∗^ *p* < 0.001	0.40^∗∗^ *p* < 0.001	1	0.34^∗∗^ *p* < 0.001	0.19^∗^ *p* = 0.011	0.17^∗^ *p* = 0.027
Language ability	−0.60^∗∗^ *p* < 0.001	0.45^∗∗^ *p* < 0.001	0.34^∗∗^ *p* < 0.001	1	0.44^∗∗^ *p* < 0.001	0.58^∗∗^ *p* < 0.001
Non-verbal IQ	−0.270^∗∗^ *p* < 0.001	0.16^∗^ *p* = 0.037	0.19^∗^ *p* = 0.011	0.44^∗∗^ *p* < 0.001	1	0.29^∗∗^ *p* < 0.001
Memory span	−0.33^∗∗^ *p* < 0.001	0.23^∗∗^ *p* = 0.002	0.17^∗^ *p* = 0.027	0.58^∗∗^ *p* < 0.001	0.29^∗∗^ *p* < 0.001	1

*^∗^*p* < 0.05, ^∗∗^*p* < 0.01. Speech-in-noise perception measured by age-adjusted HINT SRT in noise [dB SNR)]; speech perception (sentences) measured by sentences in quiet (% words correct); speech perception (monosyllables) measured by % correct words; language ability measured by The Core Language Index of the Clinical Evaluation of Language Fundamentals (CELF-4) (standard score); non-verbal IQ measured by Raven’s matrices (standard score); memory span measured by CELF Digit span subtest (scaled score of forward and backward digit span combined).*

**TABLE 4:** Linear model of predictors of speech-in-noise perception for the full group of participants (*n* = 175).

	***B* [95% CI]**	***SE* B**	**β**	***p***
Constant	12.26 [7.85, 16.7]	2.23		<0.001
Group difference	HA: 1.54 [0.57, 2.51]	0.49	0.19	0.002
from TD group	DLD: −0.29 [−1.57, 0.99]	0.65	-0.03	0.66
	CI: 2.87 [1.91, 3.83]	0.49	0.43	<0.001
Language ability	−0.057 [−0.075, −0.039]	0.009	−0.36	<0.001
Speech perception (monosyllables)	−0.094 [−0.14, −0.052]	0.021	−0.26	<0.001
Non-verbal IQ	−0.007 [−0.031, 0.016]	0.012	−0.031	0.550
Memory span	0.027 [−0.12, 0.17]	0.075	0.021	0.720
Adjusted *R*^2^ = 0.64				

*Forced entry method of regression was used.*

**FIGURE 3:**
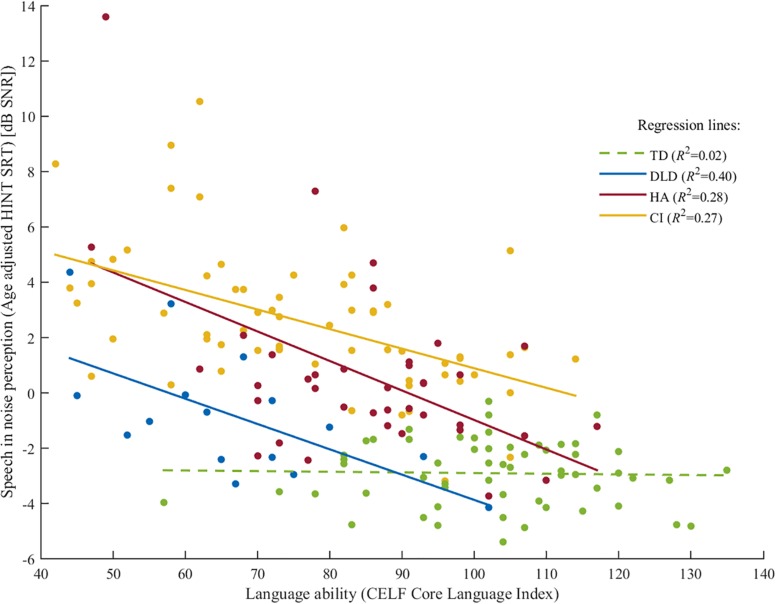
Scatterplot of HINT SRTs versus CELF-4 Core Language Index for children with CI (yellow), HA (red), DLD (blue) and TD (green). The solid lines are linear regression lines for groups CI, HA, and DLD (*p* ≤ 0.005). The green dashed line is a non-significant linear regression line (*p* = 0.29) for the TD group.

To further investigate the relationship between language ability and SiN perception, the data points were plotted in a scatter plot where each point represented an individual’s language ability on the *x*-axis and SiN perception on the *y*-axis. Figure 3 shows a scatterplot of SiN perception versus language ability with regression lines for the four groups. The scatterplot shows that SiN perception scores for the CI group were poorer than for the DLD group, but both groups showed a small-medium linear effect of language ability on SiN perception. The HA group showed a similar linear effect. However, the regression line may have been influenced by two outliers as described in section “Factors That Predict Speech in Noise Perception in Children With HA.” The regression line for the TD group showed 0 slope which means that there was little to no effect of language ability on SiN perception. The scatterplot also indicates that language ability had more effect on speech perception in noise when language ability was lower than approximately 85, which is −1 SD below the normative mean, compared to when language ability was above 85. However, when modeling SiN perception in the linear regression model, we made the assumption that language ability linearly predicted SiN perception in the language ability interval of interest (from 42 to 135).

#### Factors That Predict Speech in Noise Perception in Children With CIs

For the CI group, the variable age at implantation was investigated in addition to the other independent variables for the subgroup of children who had not acquired oral language before implantation (see description in section Characteristics of the Children in the CI Group). Table 5 reports correlations among variables for the whole CI group, and in addition correlation with implantation age for the subgroup who had not acquired oral language before implantation. In the full CI group, all variables except non-verbal IQ and memory span were significantly correlated with SiN perception.

**TABLE 5:** Correlations among variables for children with CI (*n* = 64) and correlations with implantation age for subgroup who had not acquired language before implantation (*n* = 46).

	**Speech in noise perception (SiN)**	**Speech perception (sentences)**	**Speech perception (monosyllables)**	**Language ability**	**Non-verbal IQ**	**Memory span**
Speech in noise perception (SiN)	1	−0.67^∗∗^ *p* < 0.001	−0.28^∗^ *p* = 0.03	−0.52^∗∗^ *p* < 0.001	−0.06 *p* = 0.63	−0.12 *p* = 0.34
Speech perception (sentences)	−0.67^∗∗^ *p* < 0.001	1	0.13 *p* = 0.30	0.51^∗∗^ *p* < 0.001	0.07 *p* = 0.61	0.08 *p* = 0.54
Speech perception (monosyllables)	−0.28^∗^ *p* = 0.03	0.13 *p* = 0.30	1	0.07 *p* = 0.57	−0.057 *p* = 0.66	−0.13 *p* = 0.32
Language ability	−0.52^∗∗^ *p* < 0.001	0.51^∗∗^ *p* < 0.001	0.07 *p* = 0.57	1	0.35^∗∗^ *p* = 0.006	0.52^∗∗^ *p* < 0.001
Non-verbal IQ	−0.06 *p* = 0.63	0.07 *p* = 0.61	−0.057 *p* = 0.66	0.35^∗∗^ *p* = 0.006	1	0.37^∗∗^ *p* = 0.003
Memory span	−0.12 *p* = 0.34	0.08 *p* = 0.54	−0.13 *p* = 0.32	0.52^∗∗^ *p* < 0.001	0.37^∗∗^ *p* = 0.003	1
Implantation age (*n* = 46)	0.40^∗∗^ *p* = 0.006	−0.08 *p* = 0.59	−0.04 *p* = 0.77	−0.50^∗∗^ *p* < 0.001	−0.31^∗^ *p* = 0.04	−0.30 *p* = 0.04^∗^

*^∗^*p* < 0.05, ^∗∗^*p* < 0.01.*

A model was fitted for the CI group with SiN perception as the dependent variable including all the five independent variables. When non-verbal IQ and memory span were removed from the model (*p*-values 0.5 and 0.9 respectively) the model explained one percentage point less of the variance. Table 6 shows the results of the latter multiple linear regression model which accounted for 50% of the variance. For the subgroup who had not acquired language before implantation, the same predictors were used in a model together with implantation age. This model is also reported in Table 6, and accounted for 55% of the variance in SiN.

**TABLE 6:** Linear model of predictors of speech in noise perception for children with CI.

	***B* [95% CI]**	***SE B***	**β**	***p***
**CI participants, whole group (*n* = 64)**				
Constant	33.04 [23.91, 42.17]	4.47		<0.001
Speech perception (monosyllables)	−0.069 [−0.13, −0.01]	0.03	−0.20	0.033
Speech perception (sentences)	−0.23 [−0.32, −0.14]	0.05	−0.52	<0.001
Language ability	−0.033 [−0.06, −0.01]	0.01	−0.25	0.021
Adjusted *R*^2^ = 0.50				

**CI participants, subgroup who had not acquired language before implantation (*n* = 46)**				
Constant	30.71 [6.00, 21.17]	4.74		0.001
Implantation age	0.83 [−0.24, 1.23]	0.30	0.33	0.009
Speech perception (monosyllables)	−0.074 [−0.17, −0.02]	0.03	−0.57	0.021
Speech perception (sentences)	−0.24 [−0.32, −0.14]	0.05	−0.34	<0.001
Language ability	−0.005 [−0.086, −0.013]	0.02	−0.04	0.782
Adjusted *R*^2^ = 0.55				

*Forced entry method of regression was used.*

There were significant correlations between some of the independent variables, e.g., between language ability and both non-verbal IQ and memory span (see Table 5), but correlations were weak to moderate, and the variance inflation factors for all predictors were below 2.1, which suggest no threat of multicolinearity (Hair et al., 1995). Diagnostic statistics revealed one potential influential data point. This data point had large leverage and Mahalanobis distance values, suggesting undue influence on the model (Barnett and Lewis, 1978; Stevens, 2002). Cook and Weisberg (1982) suggest that a Cook’s distance value greater than 1 is of concern. This data point did not exceed 1. Further inspection of this data point revealed that this child had a low language ability score. However, this data point was not an outlier, i.e., not smaller than 1.5 times the interquartile range, and removing this data point did not substantially change the coefficients or the significance of the predictors. Without the data point the model accounted for 4 percentage points less of the variance. The data point was thus included in the multiple linear regression model presented in Table 6.

#### Factors That Predict Speech in Noise Perception in Children With HA

Table 7 reports correlations among all variables for children with HA. All variables except memory span correlated with SiN perception. However, all variables were initially included in a regression model which accounted for 59% of the variance with speech perception in quiet as the only significant predictor. When non-verbal IQ and memory span were removed from the regression analyses, the model accounted for 3 percentage points less of the variance. In addition, the unique variance explained by speech perception in quiet decreased by 3%, while the unique variance explained by speech perception in quiet (monosyllables) and language ability increased by 9 and 7% respectively. Table 8 shows the results of the multiple linear regression model of SiN perception with speech perception in quiet (monosyllables), speech perception in quiet (sentences), and language ability as predictors. The model accounted for 52% of the variance. All three predictors significantly influenced the model.

**TABLE 7:** Correlations among variables for children with HA (*n* = 37).

	**Speech in noise perception (SiN)**	**Speech perception (sentences)**	**Speech perception (monosyllables)**	**Language ability**	**Non-verbal IQ**	**Memory span**
Speech in noise perception (SiN)	1	−0.66^∗∗^ *p* < 0.001	−0.46^∗∗^ *p* = 0.004	−0.53^∗∗^ *p* = 0.001	−0.41^∗^ *p* = 0.011	−0.23 *p* = 0.18
Speech perception (sentences)	−0.66^∗∗^ *p* < 0.001	1	0.36^∗^ *p* = 0.028	0.53^∗∗^ *p* = 0.001	0.19 *p* = 0.25	0.14 *p* = 0.40
Speech perception (monosyllables)	−0.46^∗∗^ *p* = 0.004	0.36^∗^ *p* = 0.028	1	0.071 *p* = 0.68	0.36^∗^ *p* = 0.027	−0.025 *p* = 0.89
Language ability	−0.53^∗∗^ *p* = 0.001	0.53^∗∗^ *p* = 0.001	0.071 *p* = 0.68	1	0.42^∗∗^ *p* = 0.009	0.47^∗∗^ *p* = 0.003
Non-verbal IQ	−0.41^∗^ *p* = 0.011	0.19 *p* = 0.25	0.36^∗^ *p* = 0.027	0.42^∗∗^ *p* = 0.009	1	0.32 *p* = 0.053
Memory span	−0.23 *p* = 0.18	0.14 *p* = 0.40	−0.025 *p* = 0.89	0.47^∗∗^ *p* = 0.003	0.32 *p* = 0.053	1

*^∗^*p* < 0.05, ^∗∗^*p* < 0.01.*

**TABLE 8:** Linear model of predictors of speech in noise perception for children with HA.

	***B* [95% CI]**	***SE B***	**β**	***p***
**HA group (*n* = 37)**				
Constant	32.99 [4.29,42.04]	5.95		0.001
Speech perception (sentences)	−0.21 [−0.38,0.03]	0.08	−0.39	0.012
Speech perception (monosyllables)	−0.085 [−0.15,0.04]	0.04	−0.30	0.024
Language ability	−0.061 [−0.11,−0.00]	0.03	−0.30	0.035
Adjusted *R*^2^ = 0.52				

**Two influential points excluded (*n* = 35)**				
Constant	17.09 [0.30,30.84]	7.69		0.032
Speech perception (sentences)	−0.027 [−0.23,0.07]	0.10	−0.05	0.781
Speech perception (monosyllables)	−0.073 [−0.18,0.08]	0.03	−0.36	0.037
Language ability	−0.046 [−0.11,0.00]	0.03	−0.28	0.104
Adjusted *R*^2^ = 0.13				

*Forced entry method of regression was used.*

There were significant correlations between some of the independent variables, however only a positive weak to moderate correlation for language ability and both non-verbal IQ and memory span (see Table 7), and the variance inflation factors for all predictors were below 2.1 (largest and equal to 2.1 for language ability), which suggest no threat of multicolinearity (Hair et al., 1995).

Diagnostic statistics revealed two potential influential data points. Both data points had large leverage and Mahalanobis distance values. Further inspection showed that one child had a very low monosyllable perception score (48%, which was an outlier) despite having a good speech perception in quiet score for sentences (98%). The other child had very low scores for monosyllable perception (64%), speech perception in quiet (70%), language ability (49) and required a very high SRT (13.6 dB SNR) for SiN perception - all of these scores were outliers. The latter child had a value of Cook’s distance that exceeded 1, suggesting undue influence on the model (Cook and Weisberg, 1982). When both of these data points were removed the predictors were no longer significant and the model accounted for only 13% of the variance. Table 8 shows the impact of removing the two influential points on the regression model.

#### Factors That Predict Speech in Noise Perception in Children With DLD

Table 9 reports correlations among all variables for the DLD group. SiN perception was strongly related to language ability, but not significantly related to non-verbal IQ or memory span. There was little variation in the measures of speech perception of sentences and monosyllables in quiet [see Table 1 and descriptions in sections Speech Perception in Quiet (Monosyllables) for Children With CI, HA, DLD, and TD and Speech Perception in Quiet (Sentences) for Children With CI, HA, DLD, and TD]. The ceiling effect and the small variance in results on these two tests prohibited their use as predictors in the regression model.

**TABLE 9:** Correlations among variables for group with DLD (*n* = 16).

	**Speech in noise perception (SiN)**	**Speech perception (sentences)**	**Speech perception (monosyllables)**	**Language ability**	**Non-verbal IQ**	**Memory span**
Speech in noise perception (SiN)	1	−0.42 *p* = 0.11	−0.11 *p* = 0.69	−0.63^∗∗^ *p* = 0.009	−0.38 *p* = 0.15	−0.37 *p* = 0.16
Speech perception (sentences)	−0.42 *p* = 0.11	1	0.86^∗∗^ *p* < 0.001	0.38 *p* = 0.15	0.13 *p* = 0.62	0.34 *p* = 0.20
Speech perception (monosyllables)	−0.11 *p* = 0.69	0.86^∗∗^ *p* < 0.001	1	0.32 *p* = 0.23	0.11 *p* = 0.69	0.43 0.099
Language ability	−0.63^∗∗^ *p* = 0.009	0.38 *p* = 0.15	0.32 *p* = 0.23	1	0.62^∗^ *p* = 0.011	0.61^∗^ *p* = 0.013
Non-verbal IQ	−0.38 *p* = 0.15	0.13 *p* = 0.62	0.11 *p* = 0.69	0.62^∗^ *p* = 0.011	1	0.47 *p* = 0.067
Memory span	−0.37 *p* = 0.16	0.34 *p* = 0.20	0.43 0.099	0.61^∗^ *p* = 0.013	0.47 *p* = 0.067	1

*^∗^*p* < 0.05, ^∗∗^*p* < 0.01.*

Due to the small sample size, multiple regression was not conducted for the DLD group. Simple linear regression was carried out to investigate the relationship between SiN perception and language ability. There was a significant relationship between SiN perception and language ability with slope equal to −0.09 dB per unit change in language ability (*p* = 0.009), and 40% of the variance in SiN perception could be explained by the model containing only language ability. There were two children with DLD who did not have ceiling scores for monosyllable perception and sentence repetition in quiet (the same two children had low scores for both tests). When these two children were removed from the regression analysis, there was still a significant relation between SiN perception and language ability with slope equal to −0.0.07 (*p* = 0.03), and 33% of the variance in SiN perception was explained by the model containing only language ability.

#### Factors That Predict Speech in Noise Perception in Children With TD

Table 10 reports correlations among all variables for the TD group. None of the variables correlated significantly with SiN perception (all had *p* > = 0.25), and thus we did not carry out a regression analysis for the TD group. As Table 1 shows, there was little variation in speech perception in quiet (monosyllable perception and sentence repetition). Therefore interpretation of correlations is valid only within the very limited range of values for these two scores. For the TD group, all except three participants had language ability standard scores above 80, and thus it should be kept in mind that the non-significant relationship between language ability and SiN perception was observed in this range of normal language ability.

**TABLE 10:** Correlations among variables for group with TD (*n* = 58).

	**Speech in noise perception (SiN)**	**Speech perception (sentences)**	**Speech perception (monosyllables)**	**Language ability**	**Non-verbal IQ**	**Memory span**
Speech in noise perception (SiN)	1	0.15 *p* = 0.25	−0.11 *p* = 0.40	−0.03 *p* = 0.83	−0.14 *p* = 0.29	0.08 *p* = 0.58
Speech perception (sentences)	0.15 *p* = 0.25	1	0.13 *p* = 0.32	0.28^∗^ *p* = 0.034	−0.05 *p* = 0.70	0.02 *p* = 0.91
Speech perception (monosyllables)	−0.11 *p* = 0.40	0.13 *p* = 0.32	1	0.18 *p* = 0.18	−0.04 *p* = 0.77	−0.04 *p* = 0.79
Language ability	−0.03 *p* = 0.83	0.28^∗^ *p* = 0.034	0.18 *p* = 0.18	1	0.32^∗^ *p* = 0.02	0.23 *p* = 0.08
Non-verbal IQ	−0.14 *p* = 0.29	−0.05 *p* = 0.70	−0.04 *p* = 0.77	0.32^∗^ *p* = 0.02	1	−0.09 *p* = 0.53
Memory span	0.08 *p* = 0.58	0.02 *p* = 0.91	−0.04 *p* = 0.79	0.23 *p* = 0.08	−0.09 *p* = 0.53	1

*^∗^*p* < 0.05.*

#### Developmental Trend of Speech in Noise Perception in Children With TD

The HINT SRTs were corrected for (i) the acoustic environment, i.e., anechoic chamber or audiometric test room, and (ii) age (5;6–10;5 years) using the regression equation from Myhrum et al. (2016) with slope −0.69 dB/annum and with 95% CI (−0.84, −0.55). There is some evidence to suggest that children reach adult performance on the Norwegian HINT by 9–10 years of age in test conditions where speech and noise are collocated in front of the listener (Myhrum et al., 2016). However, the ages at which adult performance is reached vary slightly in similar studies using other HINT languages. For the HINT versions for American English (Nilsson et al., 1996) and French Canadian (Vaillancourt et al., 2008), significant differences were found between 10-year-olds and adults (1.5 and 1.0 dB SNR respectively), but not between 12-year-olds and adults, indicating that adult performance was reached between 10 and 12 years of age. In a study using the Words-in-noise test (with monosyllabic words as stimuli) Wilson et al. (2010) found that the recognition performance was stable between ages 9 and 12 years.

To investigate the age effect on HINT in the TD group in the current study, we examined the uncorrected HINT data, calculating the linear regression of HINT versus age. The slope of the HINT versus age regression for TD children of ages (5;6–10;5 years) was equal to −0.57 dB/annum (*p* < 0.001, explaining 29% of the variance). This is close to the slope used for the age norm correction (−0.69), and is within the confidence interval of the slope estimated in Myhrum et al. (2016). A second finding was that the slope of HINT versus age when including the children above 10 years (5;6–12;5 years) was less steep (−0.36). Furthermore, in the current study, the mean HINT SRTs for 10–12-year-olds in the TD group were approximately the same (10 years: *n* = 6, *m* = −2.76 dB, 11 years: *n* = 9, *m* = −2.19 dB, 12 years: *n* = 4, *m* = −2.49 dB). These sample sizes are too small to draw robust conclusions, but support claims that the developmental trend seen in the Norwegian HINT perception trails off by 10 years of age.

The age effect was further investigated by calculating the correlation between the age-adjusted HINT SRTs and age in the TD group. We found a weak positive correlation (*r* = 0.28, *p* = 0.03). A linear regression between age and the age-adjusted HINT gave a slope equal to 0.16 dB/annum. This weak positive correlation may indicate that in the TD group, the HINT SRTs of the younger children may have been somewhat overcorrected by applying the norm reported in Myhrum et al. (2016).

## Discussion

The present study investigated SiN perception in four groups of children: children with CI, HA, DLD, or TD. We aimed to identify the differences in performance on the HINT and to investigate which cognitive and linguistic factors predict SiN perception for these children.

### Group Differences in Speech-in-Noise Perception

As we would expect, children with TD had, on average, the best SiN perception. There was a reliable difference between all groups in SiN perception ability except between the HA and DLD groups. Consistent with past literature, these findings show that children with permanent hearing loss and DLD exhibit speech perception deficits in noise (Ziegler et al., 2011; Misurelli and Litovsky, 2012; Nittrouer et al., 2013; McCreery et al., 2015). The finding that children with HA and CI require a higher SNR to perceive speech in noise should encourage educational settings to improve the SNR in the environment for these children. Through assistive listening device technology such as FM radio signal, infrared light, and induction loop systems, children with HA and CI can better access speech in background noise. For children who do not use personal hearing devices, like children in the DLD and TD groups, classroom sound field systems can be used to help them listen in less-than-ideal conditions.

It should be kept in mind that the CI group included in the present study was not representative of the pediatric CI population as a whole, but was composed of those children who had relatively good speech perception in quiet. Thus, differences between children with CI and the other three groups, including children with HA, would likely have been substantially larger if children with CI with poorer performance on speech perception tasks in quiet had also been included. However, inclusion of this group of children with CI would have required the use of a different test to measure SiN perception, as the HINT standard adaptive procedure would likely have been too demanding.

While the DLD group was too small (*n* = 16) to draw robust conclusions, the findings represent preliminary evidence that some children with DLD may underperform not only on SiN perception tasks that assess fine phonological contrasts through monosyllable repetition (Ziegler et al., 2005, 2011), but also tasks that use simple sentences as stimuli. Our results differ from those of Ferguson et al. (2011) who found no difference between children with DLD and TD peers on a sentence repetition in noise task. One possible reason for this difference may be the scoring method. The sentences used by Ferguson et al. (2011) were based on materials and a scoring method reported by MacLeod and Summerfield (1990). Three of the words in each sentence were designated as keywords, and a correct score was given if these keywords were repeated. By contrast, in the present study, it was required that all words in the sentence (approximately 5 on average) were repeated correctly. Although almost all children with DLD were at ceiling when repeating the HINT sentences in quiet, the double demands of noisy conditions and the number of words that had to be repeated may have contributed to the deficit compared to TD peers on this task.

### Factors Predicting Speech-in-Noise Perception

Whereas there was no relation between SiN perception and language ability in the TD group, language ability predicted unique variance in SiN perception for children in all three clinical groups, even when speech perception in quiet (monosyllable perception) was taken into account. This finding of a relation between SiN perception and language ability is in line with previous work on children with hearing loss (e.g., Ching et al., 2018). While the current study cannot determine the mechanisms responsible for the relation between SiN performance and language ability, we can think of several possible reasons for the observed association. One possibility is that the language demands posed by the HINT sentences were simply too high for the children with CI, HA, and DLD, despite efforts to keep the stimulus sentences at a level that was easily comprehensible and repeatable for 5-year-olds. However, approximately 90% of children in the HA and CI groups had a score of 90% correct or more on the sentence repetition task in quiet, and only two out of 16 children with DLD had below-ceiling performance in quiet. Thus, it appears that for the great majority of children in all three clinical groups, the vocabulary, grammar and length of the HINT sentences were manageable under optimal listening conditions.

However, it is possible that more difficult listening conditions involving noise require more robust linguistic knowledge, as the matching between input and linguistic memory representations has to be completed with only partial information. If phonological or lexical representations are less detailed or unstable in children with hearing loss or DLD, or the ability to suppress competing lexical candidates is deficient, the degraded auditory input may not be sufficient for activating the correct lexical items. Grammatical skills may also be needed to supplement word recognition under difficult conditions, e.g., by proving information about the likely word class of an upcoming word. Additionally, if recognition processes are not able to settle on a word, this may have cascading effects for recognition of subsequent words in the speech stream. Thus, children with hearing loss and DLD may have language representations and processing mechanisms which suffice in optimal conditions (with simple sentences), but which are not robust enough to support efficient SiN perception. This interpretation is in line with previous studies suggesting that language knowledge can counteract the consequences of deficits in supra-threshold auditory processing tasks as it allows participants to better ‘guess’ the words in the sentences based on regularities and context (Bradlow et al., 2003; Sperling et al., 2005; Conway et al., 2014).

Another possible explanation for the observed association between SiN perception and language ability in children with hearing loss may be that children who have hearing-in-noise deficits get less and poorer quality language input in a number of everyday situations which are typically noisy, such as preschool and school, and therefore pick up less language. In other words, the hearing-in-noise deficit may be a cause of poor language skills. The difference in input between children with hearing loss and peers with typical hearing may be especially prominent in third-party learning situations, i.e., when the language is not addressed directly to the child, but to another person in the child’s surroundings. A number of experimental studies of TD children show that they can learn words through listening in on others’ conversations (for a review, see Akhtar et al., 2019), but this may be substantially more difficult for children with hearing loss, and especially under noisy conditions.

Although the language deficit displayed by children with CI or HA in this study may be traced back to poor audibility and phonetic discrimination, it still appears to pose an additional challenge when attending to speech in noise. Nittrouer et al. (2013) found that phonological sensitivity explained a significant amount of between-groups variance in SiN perception for children with HA, children with CI and children with typical hearing, and thus conclude that “it is not enough to focus only on ways to improve the acoustic environment; their language abilities also must be considered” (p. 523). Our findings are consistent with the view that interventions designed to help children with hearing loss develop good language skills could potentially be an effective way to improve their capabilities to handle noisy school environments. Examination of whether gains in language skills resulting from language intervention are coupled with gains in SiN perception could also help determine whether better language skills are causally related to better SiN perception.

A clinical implication of the robust relation between HINT performance and language ability in children with hearing loss and DLD is that a full interpretation of HINT results for children in these groups should be made in conjunction with an assessment of the child’s language skills.

The fact that language ability was not a significant predictor of SiN for the TD children in the present study may suggest that the linguistic load of the HINT sentences was low, even in demanding processing conditions, for TD school age children. However, language ability has been shown to predict speech perception in noise in other studies of normal hearing participants. For example, in a study by MacCutcheon et al. (2019), SiN perception was better in the 50% of participants who had the best expressive language scores. As sentence repetition is one of the best measures of individual differences in language ability (Klem et al., 2015), it is possible that language ability would have come out as a significant predictor also in the present study if sentences had been linguistically more challenging, e.g., using less frequent words or complex syntax.

For the full sample of participants, there was a moderate and significant correlation between SiN perception and memory span, as measured by the composite of forward and backward digit span, but this correlation was only about half the effect size of the correlation between SiN and language ability. Memory span was not a significant predictor of SiN perception when language ability and speech perception in quiet were taken into account. The finding of a significant association between memory span and SiN is in line with a number of previous studies of children (MacCutcheon et al., 2018, 2019, submitted). Still, our results suggest that for children with hearing loss and language disorder, general language ability may be more closely related to SiN perception. This is evidenced by the fact that for children with CI, HA and DLD, when seen as separate groups, there was a strong and significant bivariate correlation between SiN perception and language ability, but no significant correlation with memory span. This pattern of findings may partly be due to the language measure being more robust, as it represents a composite score from four comprehensive subtests, while the memory span was composed only of two subtests. Another possible reason for the relatively weak relationship between memory span and SiN performance in the present study, was that the sentences used in the HINT are relatively short. Moreover, the sentences mostly describe well known scenarios and thus allow the participant to use linguistic context and world knowledge to compensate for memory limitations. By contrast, the SiN task used by MacCutcheon et al. (2018; 2019, submitted) where all sentences follow the same template with some items (colors and numbers) replaced in each sentence, does not allow for use of linguistic context or world knowledge.

As expected, speech perception in quiet, measured by sentence repetition and monosyllable repetition, was related to SiN performance for children with CI and HA. However, most children, even in the two groups of children with hearing loss, had near ceiling performance on the HINT sentence repetition test. For children with CI and HA monosyllable repetition scores had a bell-shaped distribution around the average score of 87–88%, indicating that even if sentences could be repeated without errors, discrimination of monosyllabic words without a linguistic context was challenging. The ceiling effects for the monosyllable repetition in the TD and DLD groups can be explained by the fact that real (and frequent) words were used. Had non-words been used instead of real words, performance on these tests would possibly have explained more variance in SiN performance, especially for younger children.

For the subgroup of children who had a congenitally profound to severe hearing loss and who did not acquire spoken language before CI (*n* = 46), implantation age significantly predicted SiN perception above and beyond speech perception in quiet scores. This finding is in line with evidence from previous research on SiN perception in children with CI (Ching et al., 2018).

When using HINT SRTs which were not corrected for age, age was a significant predictor of speech perception in noise for the TD children, and the estimated developmental trend was quite similar to the developmental trend estimated in the paper presenting the Norwegian HINT normative data (Myhrum et al., 2016). The developmental trend in SiN perception is also consistent with previous research (Jamieson et al., 2004; Neuman et al., 2010; Wilson et al., 2010).

### Limitations and Future Directions

In the current study we investigated predictors of SiN perception both in the full sample of 175 children and separately for each of the four participant groups. While the group-specific analyses were important for comparisons with previous studies of these groups, it should be acknowledged that a large number of statistical comparisons were carried out, thus increasing the probability of erroneous inferences. In addition, the study spanned a wide age-range (from 5;6 to 12;11 years), and while age norms were used, these norms may have been better suited for children with TD than for clinical samples, as the norming samples typically have few children at the lower tail of the distribution. The DLD sample in the present study was small, and thus we cannot draw robust conclusions about SiN perception in this group.

Another limitation of the study was the ceiling effects on the tests of speech perception in quiet in the TD, DLD (ceiling effects for both sentences and monosyllables) and, to some extent the HA and CI groups (ceiling effects for sentences), which made it difficult to assess the predictive value of speech perception in quiet for SiN perception. A nonsense word repetition test may have given a more fine-grained and better distributed measure of speech perception in quiet (for an overview of advantages of nonsense word repetition tests to assess speech perception, see Rødvik et al., 2018). Additionally, we only used OAEs (in combination with parent report of normal hearing) to assess hearing in the DLD and TD groups. OAEs do not give precise information of hearing thresholds. Thus, it is possible that subclinical differences in audiometric thresholds may have explained some of the observed variance within the two normal-hearing groups.

A limitation which applies to the CI and HA groups especially, was that the language ability and memory span tests were administered in the auditory modality. Although the tests were given in a quiet one-to-one setting, listening effort have likely been higher for the children with hearing loss (for a discussion of the interaction of perceptual and cognitive load, see e.g., Rönnberg et al., 2010). Listening effort may in turn have affected problem solving capacity and possibly led to fatigue in the children with hearing loss, and thus test results may not be entirely representative for their cognitive capacity.

In the current study, we used a HINT paradigm with notionally stationary^1^ spectrally speech-shaped noise, presented from the front along with the target speech. In real world classrooms, noise will have an additional spatial and informational masking effect (for overview, see Brungart, 2001; MacCutcheon et al., 2019) as it will emanate from around the classroom and contain speech information. The SRT obtained by presenting target speech and noise from different directions would be more indicative of a real-life deficit than a score obtained for speech and noise coming from the same direction.

Furthermore, future studies could employ more ecologically valid tasks by simultaneously testing both SiN perception and another cognitive ability, e.g., by measuring differences in the outcomes of the cognitive tests when varying the difficulty of the speech perception task. Such simultaneous measures may contribute more knowledge about the interaction between deficits in SiN perception and cognitive abilities for children with hearing loss, as well as those with language disorders and typical development.

## Conclusion

Results of the present study indicate that hearing-impaired children with HA and CI, but also some normal-hearing children with DLD, struggle with spoken language perception in noise compared to normal-hearing children with TD. The measure of SiN perception that was used in the present study, the HINT, was developed to have low linguistic demands and to be appropriate for children from 5 years upwards. Still, for the children with hearing loss and language disorder in the present study, language ability explained significant variance in results, even when taking into account speech perception abilities in quiet. Results on the HINT for children with hearing loss or language disorder should therefore be interpreted in light of their language profile.

Whilst technologies, such as directional microphones and FM systems, can improve the signal-to-noise ratio and thereby improve the recognition of speech in noise for young children with hearing loss, there may also be merit for parents, teachers and clinicians in focusing on language-specific early interventions to help improve children’s capabilities to handle noisy classroom environments.

## Data Availability Statement

The datasets for this study are not publicly available at present, because a first publication from the larger project that this study is part of is currently in progress. Requests to access the datasets should be directed to OW: o.b.wie@isp.uio.no.

## Ethics Statement

The study was reviewed and approved by the Regional Committees for Medical and Health Research Ethics, South-East Norway. Written informed consent to participate was provided by the participants’ legal guardian/next of kin.

## Author Contributions

JT and OW conceived and designed the study and collected the data together with MM, research assistants, and master’s students. MM, AH, and JT performed and reported the analyses.  MM  made the figures. JT, AH, and MM wrote the manuscript with input from all authors. All authors contributed to interpreting all results and reviewing and editing the manuscript.

## Conflict of Interest

The authors declare that the research was conducted in the absence of any commercial or financial relationships that could be construed as a potential conflict of interest.

## Footnotes

1 Background noise without superimposed amplitude modulation is often referred to as a stationary or steady noise. However, Stone et al. (2011, 2012) used the term notionally steady maskers since the maskers contain random amplitude fluctuations.
